# Racial and Ethnic Disparities in Patient Experiences in the United States: 4-Year Content Analysis of Twitter

**DOI:** 10.2196/17048

**Published:** 2020-08-21

**Authors:** Yulin Hswen, Jared B Hawkins, Kara Sewalk, Gaurav Tuli, David R Williams, K Viswanath, S V Subramanian, John S Brownstein

**Affiliations:** 1 Boston Children's Hospital Boston, MA United States; 2 Department of Epidemiology and Biostatistics University of California San Francisco San Francisco, CA United States; 3 Bakar Computational Health Sciences Institute University of California San Francisco San Francisco, CA United States; 4 Innovation Program Boston Children's Hospital Boston, MA United States; 5 Computational Epidemiology Lab Harvard Medical School Boston, MA United States; 6 Department of Social and Behavioral Sciences Harvard TH Chan School of Public Health Boston, MA United States; 7 Harvard Center for Population and Development Studies Harvard University Cambridge, MA United States; 8 Center for Community-Based Research Dana-Farber Cancer Institute Boston, MA United States

**Keywords:** racial disparities, race, patient experience, policy, social media, digital epidemiology, social determinants of health, health disparities, health inequities

## Abstract

**Background:**

Racial and ethnic minority groups often face worse patient experiences compared with the general population, which is directly related to poorer health outcomes within these minority populations. Evaluation of patient experience among racial and ethnic minority groups has been difficult due to lack of representation in traditional health care surveys.

**Objective:**

This study aims to assess the feasibility of Twitter for identifying racial and ethnic disparities in patient experience across the United States from 2013 to 2016.

**Methods:**

In total, 851,973 patient experience tweets with geographic location information from the United States were collected from 2013 to 2016. Patient experience tweets included discussions related to care received in a hospital, urgent care, or any other health institution. Ordinary least squares multiple regression was used to model patient experience sentiment and racial and ethnic groups over the 2013 to 2016 period and in relation to the implementation of the Patient Protection and Affordable Care Act (ACA) in 2014.

**Results:**

Racial and ethnic distribution of users on Twitter was highly correlated with population estimates from the United States Census Bureau’s 5-year survey from 2016 (*r*^2^=0.99; *P*<.001). From 2013 to 2016, the average patient experience sentiment was highest for White patients, followed by Asian/Pacific Islander, Hispanic/Latino, and American Indian/Alaska Native patients. A reduction in negative patient experience sentiment on Twitter for all racial and ethnic groups was seen from 2013 to 2016. Twitter users who identified as Hispanic/Latino showed the greatest improvement in patient experience, with a 1.5 times greater increase (*P*<.001) than Twitter users who identified as White. Twitter users who identified as Black had the highest increase in patient experience postimplementation of the ACA (2014-2016) compared with preimplementation of the ACA (2013), and this change was 2.2 times (*P*<.001) greater than Twitter users who identified as White.

**Conclusions:**

The ACA mandated the implementation of the measurement of patient experience of care delivery. Considering that quality assessment of care is required, Twitter may offer the ability to monitor patient experiences across diverse racial and ethnic groups and inform the evaluation of health policies like the ACA.

## Introduction

In the United States, racial and ethnic minority populations experience suboptimal access to quality health care [1]. Since patient experience is strongly associated with quality health care [2-4], lower quality health care within these groups may be attributed to poorer patient experiences. Because of these poorer experiences by racial and ethnic minorities, authentic patient experience can be difficult to capture in traditional health care research. For instance, a systematic review of 44 articles showed that mistrust, stigmatization, fears, and lack of access to information prevented racial and ethnic minority groups from participating in health research [5].

Most research surrounding patient experience has been conducted by the health care system, whereby quality assessments about patient experience are retrieved from the health care institution that is providing these health care services to the patients [1,6-8]. Therefore, fear of consequences of reporting negative feedback to the health care institution that is responsible for respondents’ care may bias the results of these studies [9-11]. The validity of health care–based surveys about patient experience are put into question because a strong correlation between positive patient satisfaction and response rate has been documented [12-14]. Research suggests that levels of reported patient satisfaction with health care may be inflated due to high risks of response bias, especially among racial and ethnic minorities who have historically received poor treatment and care [13]. For example, White patients are more likely to respond to health care surveys compared with racial and ethnic minorities [15,16] and are overrepresented in many health care studies about patient experience and care [17].

Measurement of patient experience has also gained recognition as a component of the Patient Protection and Affordable Care Act (ACA) of 2010 [18]. The ACA was enacted in order to improve the access to health care through the expansion of public health coverage, improve affordability of health insurance, and make the health care system more accountable to diverse patient populations, like racial and ethnic minorities that have historically had a lack of health coverage [19]. Since the ACA’s major provisions came into force in 2014, as of 2016, it is estimated that up to 24 million additional people have received insurance coverage [20]. Additionally, the law repeatedly refers to the importance of patient-centeredness, patient satisfaction, and the measurement of patient experience of care, highlighting the importance of accurately measuring these patient-reported experiences [21,22]. Therefore, novel approaches are needed to better understand patient experience, especially among underrepresented racial and ethnic minority groups, which could also shed light on the impact of new health care policies and changing legislation, such as the implementation of the ACA.

Data derived from Twitter may offer an opportunity to capture authentic patient experiences and broaden the view of existing health care surveys on patient satisfaction. Twitter is a popular social networking and microblogging service that has over 330 million monthly active users worldwide and approximately 500 million tweets per day [23,24]. Tweets are limited to 280 characters and have been recognized as a source of organic sentiment and opinions [25]. The Twitter platform has been widely recognized as a source to monitor public sentiment across a spectrum of health-related issues, including mental health [26-28], vaccination [29], and smoking [30]. Most recently, Twitter has emerged as a potential source of information for capturing hospital and health care experiences of sexual minorities [31,32]. Considering that a greater proportion of Black and Hispanic users are on Twitter [33,34], data collected from this social media platform may make it possible to capture patient experiences from minority populations that are typically underrepresented in traditional research on patient satisfaction.

This study seeks to evaluate the ability of Twitter to monitor patient experiences in racial and ethnic minority groups in the United States. Specifically, we examined tweets about patient experience among racial and ethnic groups, including White, Black, Hispanic/Latino, Asian/Pacific Islander, and American Indian/Alaska Native patients in the United States. We further investigated trends in sentiment of these tweets across race and ethnicity from 2013 to 2016. Lastly, we explored changes in patient experience sentiment postimplementation of the ACA (2014-2016) to preimplementation of the ACA (2013) across racial and ethnic groups to understand the potential impact of this health policy on racial and ethnic disparities in patient experience.

## Methods

### Patient Experience Data Set

This study used a previously established Twitter patient experience data set to investigate racial and ethnic disparities in patient experience in the United States [35]. This patient experience data set was created from a support vector machine–based supervised machine learning classifier that was iteratively built to specifically identify tweets related to patient experience. A patient experience tweet was defined as any tweet that discussed any exposure to health care, such as care received in a hospital, urgent care, or any other health institution.

We used a geolocation inference engine validated by a previous study for the patient experience data set [35] that used a combination of users’ profiles, GPS, and the Google Maps Geocoding application programming interface to identify the geographic location of the user. A total of 2,759,257 tweets were labeled as patient experience tweets from February 1, 2013, to February 28, 2017, out of which 876,384 (31.76%) tweets were inferred to one of the 50 US states, the District of Columbia, or the US Virgin Islands by the geolocation inference engine [35]. To align our analyses with the 2016 census data, we excluded data from 2017. In total, 851,973 geolocation inference tweets were used in this analysis.

### Patient Experience Sentiment

We used natural language processing to measure the sentiment of all patient experience tweets. The sentiment of patient experience tweets was determined using a widely accepted lexicon and rule-based sentiment classifier for microblogs, Valence Aware Dictionary for Sentiment Reasoner (VADER). VADER is based on a pattern library that is trained from human-annotated words commonly found in product reviews [36,37]. VADER is often used for product reviews and news articles and therefore does not always contain similar text-based characteristics to Twitter. Therefore, we appended VADER’s dictionary and rules to provide broader representation for Twitter, such as incorporating over 110 emojis and their respective sentiment scores [38]. VADER computes sentiment for each word and generates a compound score for the sentence by summing the sentiment score of each word. We considered sentiment score to be positive if the mean compound score was greater than or equal to 0.3 and negative if the score was less than or equal to –0.3. Mean compound scores between –0.3 and 0.3 were considered neutral.

### Classification of Race and Ethnicity

The relationship between surname distribution and population structure dates to the 19th century [39,40]. More recently, the Human Genome Diversity Project has shown a strong correlation of surnames and genetic linkages between human groups [41], which has caused name-based classification of race and ethnicity to be frequently used in population-based studies to identify racial and ethnic identities when information is not directly available [39]. In 2010, the United States Census Bureau conducted a study to develop a classification system to identify racial and ethnic identities associated with a list of names from the 2010 decennial survey [42]. In this study, surnames were recorded for 295 million people (95.5% of the population) and surnames with a frequency of 100 or more were used to identify race and ethnicity. These 162,253 names cover 90% of the people recorded in the United States Census Bureau decennial survey in 2010.

Therefore, we used the United States Census Bureau surname classification system [43] to build a surname classifier to identify race and ethnicity in our Twitter population. The profile names of Twitter users were collected and matched to the United States Census Bureau surname classification database (N=162,253) to identify the race and ethnicity of Twitter users in our study [42]. Racial and ethnic categorizations were based on the categories used in the United States Census Bureau 2010 decennial survey, which included White, Black, Hispanic/Latino, Asian/Pacific Islander, and American Indian/Alaska Native persons. Out of the 851,973 geolocation tweets, 392,215 tweets were used that had racial and ethnic inferred information in the analyses presented in this study.

### Statistical Analysis

We used two methods to compare the racial and ethnic distribution of our Twitter data with the 5-year US census estimates for 2016. We first used the Kendall τ rank correlation to compare the ranking of the states by number of users that tweeted about patient experience and compared this with the rank of populations by state based on United States Census Bureau’s 2016 American Community Survey 5-year estimates. Second, we quantified the strength of the correlation of distribution across race and ethnicity for each state with a Pearson correlation test and reported the *r*^2^ coefficient variable.

We identified the relative difference in sentiment of patient experience across race and ethnicity using ordinary least squares regression. To examine the change in sentiment in patient experience across race and ethnicity from 2013 to 2016, we included a year × race/ethnicity interaction term and tested its significance in the ordinary least squares regression model. To evaluate the potential impact of the implementation of the ACA’s full provisions in 2014, we generated a dichotomous dummy variable to distinguish between the two time periods of preimplementation (2013) versus postimplementation of the ACA’s provisions (2014-2016), along with the interaction terms between that variable and the variable of race and ethnicity. This study received approval from the Boston Children Hospital’s Institutional Review Board.

## Results

### Geographic Distribution of Patient Experience Across Race and Ethnicity

**Figure 1:**
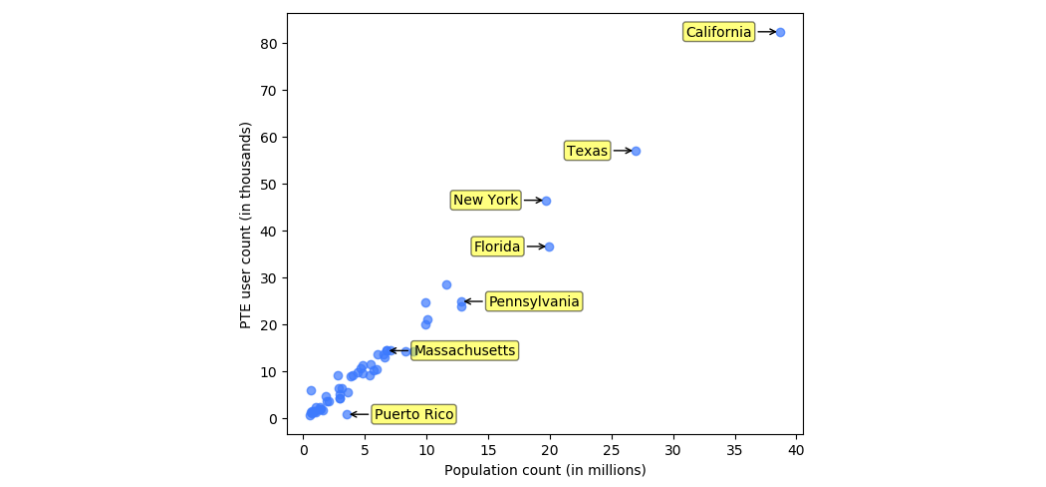
Correlation between the patient experience user counts per state and the corresponding population estimates by US census. PTE: patient experience.

**Figure 2:**
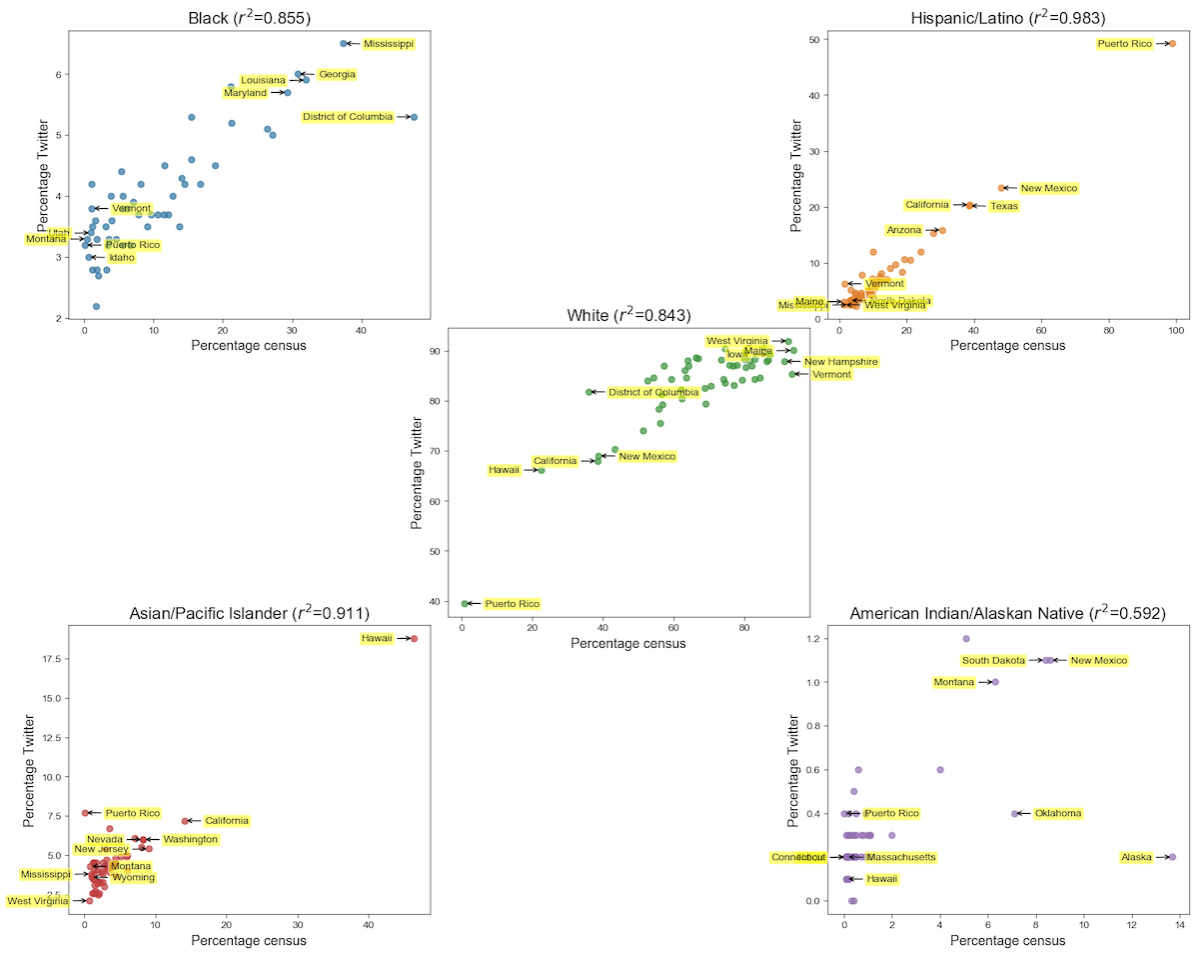
Correlation between the patient experience user counts per state and the corresponding population estimates by the US census by racial and ethnic group from 2013 to 2016.

The results between the distribution of Twitter users per state and the population estimates from the 2016 census 5-year survey show that Kendall τ was 0.845 (*P*<.001) and Pearson coefficient *r*^2^ was 0.99 (*P*<.001), as illustrated in Figure 1. The Kendall τ rank correlation was highest for the distribution of Hispanic/Latino patients (tau-b=0.74), followed by Black (tau-b=0.62), White (tau-b=0.57), Asian/Pacific Islander (tau-b=0.47), and lastly American Indian/Alaska Native patients (tau-b=0.40), with all *P* values <.001. The Pearson *r*^2^ coefficient for the correlation between the patient experience user counts per state and the corresponding population estimates by the US census was highest for Hispanic/Latino patients (*r*^2^=0.98), followed by Asian/Pacific Islander (*r*^2^=0.91), White (*r*^2^=0.85), Black (*r*^2^=0.91), and lastly American Indian/Alaska Native patients (*r*^2^=0.59), with all *P* values <.001, as seen in Figure 2.

### Patient Experience Sentiment Across Race and Ethnicity From 2013 to 2016

**Figure 3:**
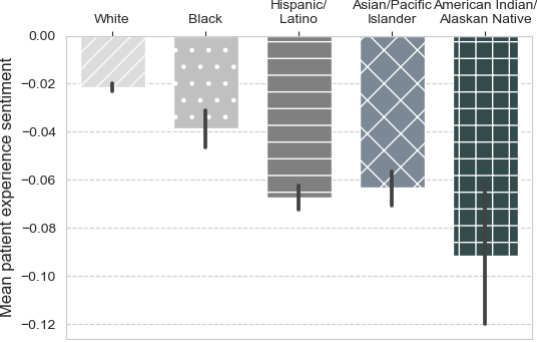
Average patient sentiment by racial and ethnic group from 2013 to 2016.

Table 1 shows the descriptive statistics of the mean, median, and standard deviation across each racial and ethnic group for each year and the overall average. The overall mean sentiment from 2013 to 2016 was highest for White patients, followed by Black, Asian/Pacific Islander, Hispanic/Latino, and lastly American Indian/Alaska Native patients, as shown graphically in Figure 3. Baseline patient experience sentiment in 2013 was the highest for White patients, followed by Black, Asian/Pacific Islander, Hispanic/Latino, and then American Indian/Alaska Native patients. In 2014, the patient experience sentiment was the highest for Black patients, followed by White, Hispanic/Latino, Asian/Pacific Islander, and then American Indian/Alaska Native patients. In 2015, the patient experience sentiment was the highest for White patients, followed by Black, Hispanic/Latino, Asian/Pacific Islander, and then American Indian/Alaska Native patients. Lastly, in 2016 the patient experience sentiment was the highest for White patients, followed by Black, Asian/Pacific Islander, Hispanic/Latino, and then American Indian/Alaska Native patients.

**Table 1:** Patient experience sentiment raw scores by racial and ethnic group from 2013 to 2016.

Race and year			Tweets, n		Sentiment, mean (SD)
**White**					
	2013	109,656		–0.043 (0.478)	
	2014	89,540		–0.018 (0.478)	
	2015	63,459		–0.004 (0.480)	
	2016	58,079		–0.005 (0.481)	
**Black**					
	2013	5552		–0.087 (0.478)	
	2014	5121		0.004 (0.489)	
	2015	3216		–0.036 (0.484)	
	2016	2766		–0.024 (0.492)	
**Hispanic/Latino**					
	2013	12,825		–0.094 (0.487)	
	2014	9495		–0.069 (0.491)	
	2015	6254		–0.037 (0.490)	
	2016	5654		–0.038 (0.492)	
**Asian/Pacific Islander**					
	2013	5734		–0.092 (0.478)	
	2014	4797		–0.059 (0.480)	
	2015	3949		–0.056 (0.500)	
	2016	3708		–0.033 (0.484)	
**American Indian/Alaskan Native**					
	2013	310		–0.154 (0.456)	
	2014	304		–0.085 (0.477)	
	2015	258		–0.078 (0.465)	
	2016	238		–0.034 (0.485)	

The linear regression model using race as the predictor variable is shown in Table 2. Compared with White patients, other racial and ethnic groups had statistically significant lower patient experience sentiments. American Indian/Alaska Native patients had the lowest patient experience sentiment, with a patient experience sentiment score that was 0.070 points less than White patients (*P*<.001). This was followed by Hispanic/Latino patients, with 0.046 fewer patient experience sentiment points (*P*<.001), then Asian/Pacific Islander patients, who had 0.042 fewer patient experience sentiment points (*P*<.001), and then Black patients, with 0.017 fewer patient experience sentiment points than White patients (*P*<.001). A graph of the average patient experience sentiment by race from 2013 to 2016 is depicted in Figure 3.

**Table 2:** Ordinary least squares modeling effects of year and racial and ethnic group on patient experience sentiment.

Predictor variables					Model 1: combined years				Model 2: yearly change					Model 3: pre-post ACA^a,b^				
					Coefficient		*P* value			Coefficient		*P* value			Coefficient		*P* value	
**Race**																		
		White			–0.0215^c^		<.001			–28.21^c^		<.001			—^d^		—	
		Black			–0.0171		<.001			–9.08		.20			—		—	
		Hispanic/Latino			–0.0460		<.001			–14.19		.01			—		—	
		Asian/Pacific Islander			–0.0419		<.001			–8.77		.18			—		—	
		American Indian/Alaska Native			–0.0702		<.001			–46.71		.08			—		—	
**Change over years 2013-2016**																		
		**Race**																
				White		—		—			0.014^c^		<.001			—		—
				Black		—		—			0.0045		.21			—		—
				Hispanic/Latino		—		—			0.0070		.01			—		—
				Asian/Pacific Islander		—		—			0.0043		.19			—		—
				American Indian/Alaska Native		—		—			0.0232		.08			—		—
**Baseline Pre-ACA (2013)**																		
	**Race**																	
			White		—		—			—		—			–0.0433^c^		<.001	
			Black		—		—			—		—			–0.0433		<.001	
			Hispanic/Latino		—		—			—		—			–0.0487		<.001	
			Asian/Pacific Islander		—		—			—		—			–0.0511		<.001	
			American Indian/Alaska Native		—		—			—		—			–0.1112		<.001	
**Change Post-ACA (2014-2017)**																		
	**Race**																	
			White		—		—			—		—			0.033^c^		<.001	
			Black		—		—			—		—			0.0388		<.001	
			Hispanic/Latino		—		—			—		—			0.0086		.28	
			Asian/Pacific Islander		—		—			—		—			0.0098		.08	
			American Indian/Alaska Native		—		—			—		—			0.0540		.09	

^a^ACA: Affordable Care Act.

^b^Change from preimplementation to postimplementation of the ACA.

^c^Reference category.

^d^Not applicable.

### Changes in Patient Experience Sentiment Across Race and Ethnicity

**Figure 4:**
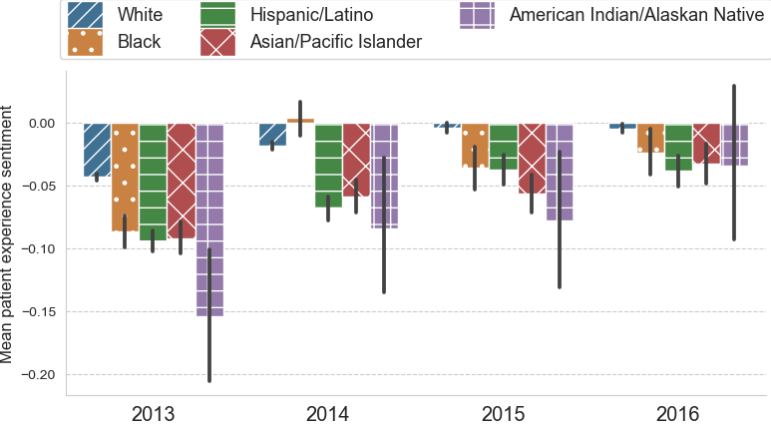
Overall average patient experience sentiment by racial and ethnic group for the years from 2013 to 2016.

The changes in slope for patient experience sentiment across each racial and ethnic group are shown in regression model 2 in Table 2 and visually displayed in Figure 4. The largest increase in mean sentiment for all racial and ethnic groups was seen from 2013 to 2014, and mean sentiment continued to increase in 2015 and 2016, as illustrated in Figure 4. The ordinary squares regression model 2 showed that the yearly increase in patient experience sentiment was 0.014 points (*P*<.001), and for Hispanic/Latino patients it was 0.021 (*P*<.001). For Black patients, it was 0.0185 points (*P*=.21), for Asian/Pacific Islander patients it was 0.0183 (*P*=.19), and for American Indian/Alaska Native patients the yearly increase was 0.037 (*P*=.08).

Patient experience increased in all racial and ethnic groups postimplementation of the ACA’s full provisions (2014-2016) compared with preimplementation of the ACA (2013), which is shown in regression model 3 in Table 2. Twitter users who identified as Black experienced a 0.0718-point (*P*<.001) change in patient experience sentiment when comparing preimplementation and postimplementation of the ACA’s full provisions. Twitter users who identified as White experienced a 0.0330-point (*P*<.001) change in patient experience sentiment from preimplementation to postimplementation of the ACA’s full provisions.

## Discussion

### Monitoring Patient Experience Sentiment of Racial and Ethnic Minority Groups Using Twitter

This study used a machine learning classifier on patient experience with data captured from Twitter to measure changes in patient experience sentiment in the United States across racial and ethnic groups. Over 2.8 million tweets about patient experience were collected and a strong correlation between the distribution of users by race and ethnicity per state and the corresponding population estimates by the US census was seen. These findings showcase a promising approach for using Twitter to capture patient health care experiences of minority racial and ethnic groups. We were also able to compare patient experiences across racial and ethnic groups and found similar disparities in patient experience that have been shown in previous patient satisfaction surveys [44,45]. Twitter users who identified as White tweeted more positively about their health care experiences compared with all other racial and ethnic groups in our study. The Hospital Consumer Assessment of Healthcare Providers and Systems, a survey overseen by the Agency for Healthcare Research and Quality that asks patients to report their health care experiences, has yielded similar results, with non-Hispanic White patients reporting better experiences compared with all other racial and ethnic groups [7]. However, this same survey found that Asian patients reported the worst disparities in hospital experiences compared with White patients [7].

There is a large absence of data from American Indian/Alaska Native patients about their experiences with health care [46]. The limited studies on American Indian/Alaska Native patients have shown that they report high levels of discrimination [47], mistrust, and low satisfaction [46]. Additionally, American Indian/Alaska Native patients have the highest health burden of illness, injury, and premature mortality [48-50]. Results from our study suggest that Twitter users who identified as American Indian/Alaska Native tweeted most negatively about their patient experience, which appears consistent with existing findings. This also indicates that Twitter may be a valuable source to capture information about this vulnerable patient group that is often difficult to capture in traditional surveys [46].

Fear about the consequences of reporting negative feedback about health care experiences is a common concern because patients who do not have good experiences with their health care system or medical providers are less likely to respond to a survey administered directly by their health care institution that provided them the care [13]. On social media platforms like Twitter, users may feel unhindered in posting content and therefore share their authentic narratives and perspectives, such as their interactions with health care services, which may be especially important in the context of experiences among racial and ethnic minorities [51]. For instance, Black Twitter users have used Twitter to foster a community network to generate public conversation about their experiences. In particular, they highlight inequities and discrimination they face [51], which can include interactions with health care services. Results from our study may overlap with this community network of Black Twitter users, whereby more negative reporting of patient experiences was observed among Black users compared with White users.

### The Affordable Care Act and Patient Experiences Across Race and Ethnicity

From 2013 to 2016, tweets about patient experience declined in negative sentiment across all racial and ethnic groups. This may suggest that users experienced fewer negative experiences in health care during this time. Twitter users who identified as Hispanic/Latino experienced a 1.5 times greater decline in negative sentiment in our study than White Twitter users, a change that may be attributed to improvements in care delivery and potentially increased access and insurance coverage within this population group between the years of 2013 and 2016. The Patient Protection and Affordable Care Act’s major provisions came into effect in 2014, and they specifically aimed to expand the quality of care and public health coverage for racial and ethnic minorities [52,53], who historically have been less likely to receive quality care and insurance compared with White patients. As a result of the implementation of the ACA, racial and ethnic minorities showed the largest gains in health insurance coverage [54-58]. From 2013 to 2016, a report from the Kaiser Family Foundation showed that Hispanic/Latino patients experienced the greatest decrease in uninsured rates compared with any other racial or ethnic group [58]. Greater insurance coverage among Hispanic/Latino patients may have led to improvement in patient experiences within this group. While it is difficult to draw conclusions from the findings in our study, it is promising that the greatest increases in patient experience sentiment on Twitter from 2013 to 2016 were observed among Hispanic/Latino patients, which parallels the reports of changing insurance coverage during this period. The Kaiser report also reported that Black and Asian/Pacific Islander patients experienced a reduction in uninsured rates, but this change was not as great as the change experienced among Hispanic/Latino patients [58].

Regression results showed that patient experience sentiment increased postimplementation of the ACA’s full provisions (2014-2016) compared with preimplementation of the ACA (2013). While our yearly regression model identified that the highest change in sentiment by year was among Hispanic/Latino patients, Twitter users who identified as Black appeared to show the most improvement in patient experience in postimplementation compared with preimplementation of the ACA’s full provisions. This change in sentiment among Black Twitter users was 2.2 times greater than the change among White Twitter users. Our results are consistent with the 2016 National Healthcare Quality and Disparities Report from the Agency for Healthcare Research and Quality, which reported a narrowing of racial and ethnic disparities in care in 2014 [59,60]. In this report, it was documented that uninsured people received worse care, but after the implementation of the Affordable Care Act’s full provisions in 2014, there was an expansion of health coverage, especially among Black and Hispanic patients [1,59,61]. This decrease in the percentage of uninsured patients within these populations appears consistent with the rise in patient experience sentiment observed in our study following the full implementation of the ACA in 2014.

### Limitations

Several limitations exist in our study. Certain populations may be excluded, which reduces the external validity of our results. Previous studies have indicated that a higher proportion of Twitter users are located in urban areas compared with rural areas, which may introduce further biases [62]. Additionally, when compared with the general population, Twitter is overrepresented by a younger population group aged 18 to 29 years [63,64], people who live in urban areas [43], and racial and ethnic minority groups [65]. On the other hand, specifically for this patient experience study, this sampling bias may have been beneficial for studying underrepresented minority populations [66,67]. Many disadvantaged groups have been shown to use social media and the internet to access health information, potentially as a means for overcoming their lack of access to adequate health care and health information [68,69]. For instance, Twitter has been used to document lesbian, gay, bisexual, transgender, and queer (LGBTQ) disparities in experience because of the higher representation of LGBTQ persons on Twitter compared with traditional surveys [31,32]. Our study used a nontraditional data set of free-forming discussions from Twitter that may capture a more authentic and truthful account of patient experiences than existing restricted hospital-based surveys, which are known to have issues of response and social desirability bias, especially among minority racial and ethnic groups [13].

We used a name-based classification system to identify race and ethnicity in this study. Therefore, our study may be limited, as this method may not have been able to fully capture race. For instance, names that could not be identified with a particular race or ethnicity using this classifier were excluded, and there is also the possibility that some names were identified incorrectly as the wrong race [39]. Additionally, name changes by marriage or for other legal reasons could not be accounted for with this system of classification. Furthermore, this name-based classification is based on preconceived race and ethnic group classifications defined by previous survey data from the United States Census Bureau’s 2010 Decennial Census. Thus, this classifier measured race and ethnicity as a single variable, which restricts the ability to depict the multifaceted nature or the complex heterogeneity found within each racial and ethnic group [70]. Lastly, our study revealed important trends in the changes in sentiment of patient experience tweets among racial and ethnic minority groups, and these paralleled similar trends related to increasing insurance coverage and access to care among minority groups following the implementation of the full provisions of the ACA in 2014. It is promising that our findings from Twitter appear consistent with these real-world changes, though it is important to recognize that our study cannot reveal whether changing conversations on Twitter are the direct result of the widespread health policy changes.

### Conclusion

Social media platforms like Twitter may provide a novel method of capturing patient experiences of racial and ethnic minority populations beyond traditional surveys. Based on our study findings on Twitter, disparities in sentiment of patient experience across race and ethnicity appear to have declined from 2014 to 2016, which parallels similar positive changes in health care services for minority groups following the implementation of the ACA’s full provisions in 2014. Continuing to monitor these patient experiences across diverse racial and ethnic groups on Twitter could be used to explore the long-term impacts of broader changes in health policies, such as the Affordable Care Act, to ensure that these disparities continue to shrink over time. Health care administrators, providers, and policy makers should consider the potential use of social media data as a method for augmenting existing measurement approaches and data sources for better understanding of patient experiences among underrepresented groups. These are particularly important steps that could inform efforts to improve the quality of care delivery among marginalized racial and ethnic minority groups.

## Abbreviations

ACA: Affordable Care Act
LGBTQ: lesbian, gay, bisexual, transgender, and queer
VADER: Valence Aware Dictionary for Sentiment Reasoner
